# Tissue-Specific Contributions to Control of T Cell Immunity

**DOI:** 10.4049/immunohorizons.2000103

**Published:** 2021-06-08

**Authors:** Amanda C. Poholek

**Affiliations:** Division of Pediatric Rheumatology, Department of Pediatrics, University of Pittsburgh, Pittsburgh, PA; and Department of Immunology, University of Pittsburgh, Pittsburgh, PA

## Abstract

T cells are critical for orchestrating appropriate adaptive immune responses and maintaining homeostasis in the face of persistent nonpathogenic Ags. T cell function is controlled in part by environmental signals received upon activation and derived from the tissue environment in which Ag is encountered. Indeed, tissue-specific environments play important roles in controlling the T cell response to Ag, and recent evidence suggests that tissue draining lymph nodes can mirror those local differences. Thus, tissue-specific immunity may begin at priming in secondary lymph nodes, where local signals have an important role in T cell fate. In this study, we discuss the tissue-specific signals that may impact T cell differentiation and function, including the microbiome, metabolism, and tissue-specific innate cell imprinting. We argue that these individual contributions create tissue-specific niches that likely play important roles in T cell differentiation and function controlling the outcome of the response to Ags.

## INTRODUCTION

Adaptive T cell immunity begins when foreign Ag activates T cells in the context of inflammatory mediators that indicate pathogenic insult to the organism has occurred. The molecular details of T cell activation have been the subject of much study for decades leading to the three-signal hypothesis that is now accepted as dogma. Furthermore, the individual contributions of TCR signaling (signal 1), costimulation (signal 2), and cytokines (signal 3) to the activation, proliferation, and differentiation of effector T cells are well understood. The eventual outcome of these three signals is transcription factor activation that both alters the transcriptome by directly turning genes on or off, as well as modifying the epigenome, which has important effects on the differentiation potential and transcriptional pathways of cells (1). The cytokine pathways that control these differentiation decisions have been well described. Briefly, type 1 responses occur in the context of IL-12, IL-27, and IFN-γ; type 2 responses in the presence of IL-4, IL-13, IL-5, and IL-9; and type 17 responses in the presence of IL-6, IL-23, and IL-1β.

In addition to responses to infection, T cells play key roles in controlling homeostasis, such as promoting regulatory T cell (Treg) populations in the presence of TGF-β, retinoic acid (RA), and IL-2 (2). Although the cytokine networks necessary to drive T cell differentiation have been well described, several studies have found alterations in the T cell cytokine response to the same Ag under different circumstances, such as the location or prior history of infection. These studies highlight the notion that the context in which an Ag is encountered plays a key role in the ultimate fate that Ag will have on T cell differentiation. Collectively, we argue that these contextual signals should be considered in addition to signals 1, 2, and 3 and that in vivo they may play key roles in shaping T cell differentiation and function.

Although much of our knowledge on T cell differentiation has been carefully worked out using in vitro assays, this non-physiological system is inherently limited and lacks many known and unknown parameters in vivo, which likely contribute to T cell activation and effector differentiation. Naive T cells are activated in the secondary lymphoid tissues, where their initial differentiation and proliferation begins. The initiation of T cell activation occurs primarily in the T cell zone via interaction with dendritic cells (DCs) (3). Activation of T cells leads to changes in chemokine receptors that drive relocalization of cells within the lymphoid tissue or allow cells to exit the lymph node (LN) and for the periphery (4). Activated CD4 T cells will migrate to the T–B border, where they can interact with Ag-presenting B cells. Successful interaction with a B cell can drive CD4 T cell differentiation into T follicular helper cells, whereas some T cells take on Th1, Th2, or Th17 programs and migrate back to the tissues to mediate effector function.

How much of the programming decisions that drive a specific effector subset occurs in the LN and how much occurs upon return to the tissue site is still unclear; however, several lines of evidence point to the notion that sufficient programming in the LN occurs to impart initial programming to an effector phenotype as well as instructions to return to the tissue space where Ag was initially encountered. This is evident in the barrier tissues such as the skin, gut, and lung, where tissue-derived factors drain to the LN and aid in programming T cells to return to that tissue. For example, DCs from the lamina propria express high levels of RALDH2, an enzyme that catalyzes the conversion of the vitamin A metabolite retinol into RA. RA-producing DCs migrate to LNs, where they drive upregulation of a4β7 and CCR9 on T cells, which are critical for homing back to the gastrointestinal tract (5–7). Similarly in the skin, local IL-1 in the dermis drives potent production of IL-17 from microbial-specific αβ T cells despite initial activation and differentiation to Th17 cells occurring in the draining LN via skin-derived DCs (8–10). Thus, although the tissues themselves are a place to direct T cell effector function, tissue-derived factors draining to the LN create an extension of the tissue space where specific instructions are imparted to T cells that shape the earliest steps of tissue-specific immunity. We argue then that although the fundamental signals required for T cell activation and differentiation are well understood, the impact of tissue-derived factors is only beginning to be appreciated for its complexity and diversity to impact T cell differentiation. In this commentary, we highlight the evidence that suggests tissues play a key role in shaping T cell responses in vivo and briefly summarize potential factors that are likely to be critical for shaping tissue-specific adaptive immune responses. Many of these topics have been more extensively covered in comprehensive reviews that we highlight; however, the goal of this perspective is to define the tissue-specific factors we believe are likely to play important roles in T cell differentiation that have been overlooked as regulators of this process.

## EVIDENCE FOR TISSUE-SPECIFIC ADAPTIVE IMMUNE RESPONSES

Although in vitro studies have elucidated the operative pathways and drivers of T cell activation and differentiation, in vivo studies provide evidence that adaptive immune responses can vary in character depending on the route of immunization or infection, suggesting tissue-specific factors play an important role in shaping the immune response. For example, bacterial infection of the respiratory tract leads to IL-17--dominated responses, whereas other routes of infection skew toward a Th1 response (11–14). The i.v. or oral infection with Listeria *monocyotgenes* produced an Ag-specific memory CD4 T cell population that produced IFN-γ upon restimulation, whereas intranasal infection promoted IL-17–producing memory CD4 T cells (15, 16). These results were in line with reports that inha-lation of *Francisella tularensis*, which is associated with more severe disease, drove increased IL-17 in comparison with the milder intradermal route of infection that promoted Th1 responses, leading to increased bacterial clearance (17). Mechanistic studies have identified several factors in the lung that support Th17 responses to infection, including PGE, TGF-β, IL-6, and IL-1β, indicating the lung environment promotes mediators that support Th17 cells that may be absent in other tissue settings (13, 14, 17). Vaccination studies have also reported that the route of immunization controls important outcomes of protection, including altered Ab isotypes produced from i.m. (IgG) or aerosol (IgA) vaccination strategies (18, 19). In contrast, systemic, intradermal, or i.m. administration drives IL-12 from DCs to promote Th1 cells (20–22). Thus, the character of the resulting T cell response can be altered, presumably because of tissue-specific factors that control the cytokine activity of innate cells present at the tissue site. Beyond bacterial infection, tissue-specific immunity is of particular importance in the gastrointestinal tract, where the constant presence of the microbiome requires regulatory control of T cell responses. T cells responding to commensals typically become Th17s or Tregs; however, in the presence of gastrointestinal infections, these responses can shift to Th1 cells, indicating that the change in environment can shape T cell differentiation (23). Responses to food Ags also promote Tregs; however, intestinal infection can shift these in a similar fashion to commensal specific responses, potentially leading to long-term consequences such as celiac disease (24–26).

In contrast, tissue-specific responses to allergens are more nuanced. Allergy is typically attributed to aberrant Th2 responses, and allergic responses occur in a wide variety of tissues, including atopic dermatitis, allergic asthma, eosinophilic esophagitis, and food allergy. Our recent study highlighted the tissue-specific nature of Th2 cell differentiation in response to allergens via the transcription factor Blimp-1. Inhaled house dust mite drove Th2 cells in the lung via a Blimp-1–dependent pathway; however, s.c. injection of house dust mite extract drove Th2 cells independently of Blimp-1 (27, 28). This was not specific to house dust mite as similar results were achieved using soluble egg Ag derived from *Schistosoma mansoni* worms. As Blimp-1 was required to indirectly promote GATA3, the master regulator of Th2 cells, these results suggested that lung-specific immune pathways occur at the point of allergen priming, possibly in the LNs draining the tissues.

**FIGURE 1.**
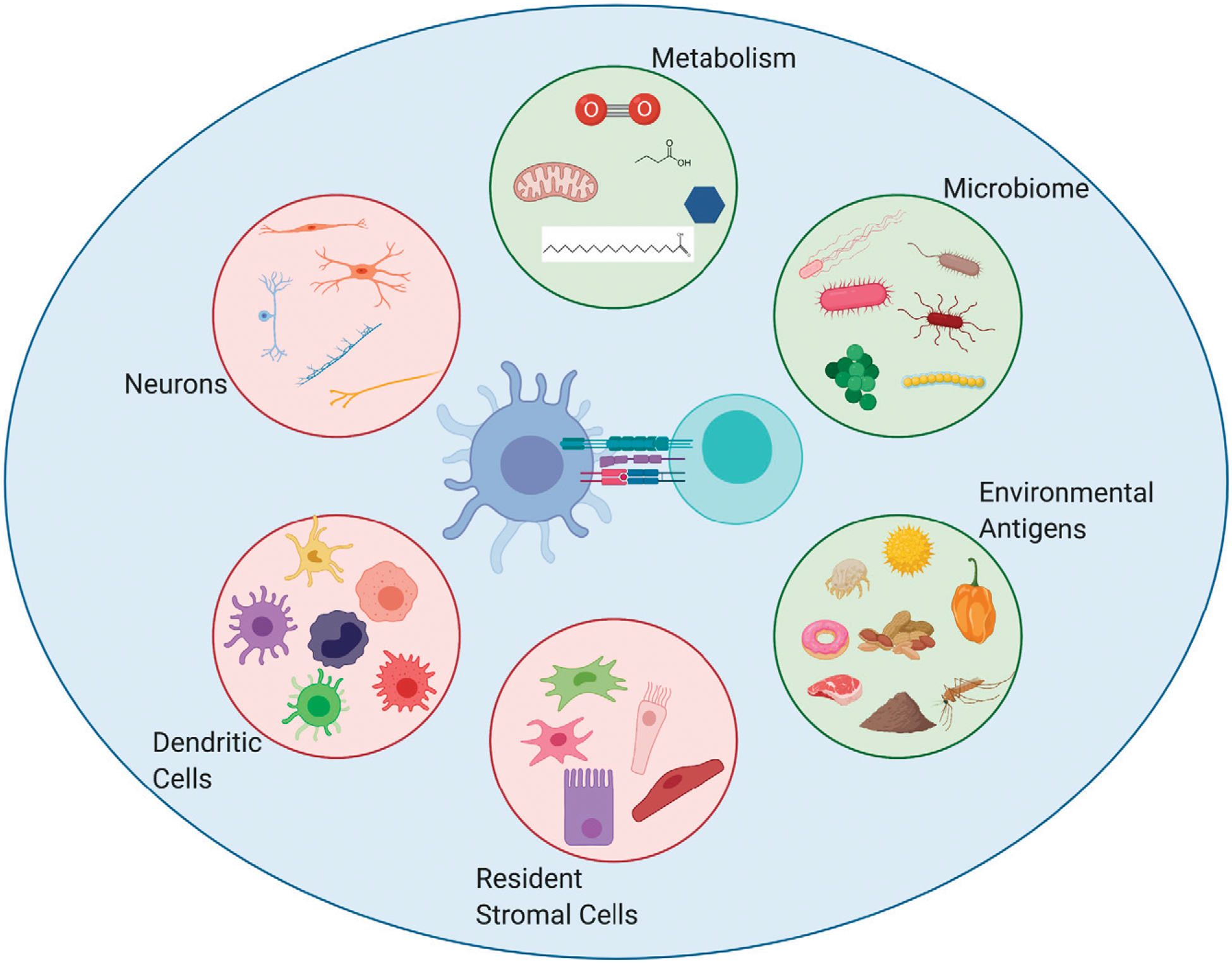
Tissue-specific factors contributing to T cell immunity. T cell differentiation is influenced by the interaction and direct signals provided by APCs but also by the surrounding environment (larger blue circle) in which it sees Ag and becomes activated. This environment can be influenced by a myriad of factors (pink and green circles), leading to alterations in T cell differentiation and function. External factors in the environment (green circles), including foreign Ags, the microbiome, and altered metabolic contexts, create tissue-specific homeostatic niches that are set early in life and can maintain a persistent state for surrounding cells, whereas cell types intrinsic to the organism (pink circles) can be modulated by the environment to promote tissue-specific contexts during homeostasis and upon infection or pathogenic insult.

Indeed, tissue immunity is not limited to the tissue space but extends to the LNs draining those tissues. Oral tolerance is critical for intestinal immunity, as the presence of food and microbial Ags creates a unique environment for the immune system to constantly survey and determine which Ags are pathogenic and which Ags require tolerance. The initiation of oral tolerance occurs in the mesenteric LNs draining the gut and requires delivery of Ag via DC transport (29). Oral tolerance is established by promoting Treg formation to commensal and food Ags because of the presence of TGF-β and RA in the gut (30–33). In line with the notion of local induction of Tregs, the propensity of T cells to form Tregs in the gut can be trans-ferred by surgical transplantation of mesenteric LNs from the gut to the popliteal fascia (34). In contrast, transfer of a peripheral LN to the mesentery only partially increased Treg formation even 8 wk posttransplant. Both the stromal compartment and the indirect effects on the DCs played a role in altering T cell differentiation toward Tregs, implicating the tissue space as playing a key role in establishing a homeostatic niche that remains in place even after transplant to a new tissue space. More recently, this idea has been extended to individual LNs that drain different portions of the gastrointestinal tract (7), where tracking responses to OVA in different gut LNs lead to alterations in the outcomes of the T cell responses (35). Surgical delivery of OVA Ag to distinct LNs lead to distinct outcomes in the percentage of Tregs or Rorγt^+^ Th17 cells formed. Furthermore, segmented filamentous bacteria (SFB)–specific Th17 cells were found in the colonic and inguinal LNs that drain regions of the gastrointestinal tract predominantly colonized by SFB, suggesting local colonization extended to the LNs draining those areas. Distinct transcriptional signatures could be found in the stromal and DC compartments from different gut LNs, suggesting that tissue-specific factors drain from the tissue site to the LN and set a homeostatic niche unique to that tissue (7, 35). Taken together, these studies provide ample evidence that T cell differentiation is impacted by the tissue in which Ag is introduced. Furthermore, the tissue environment likely extends into the local draining LN, where the homeostatic experience of the tissue can influence the differentiation of T cells upon Ag encounter either to innocuous Ag or upon infection with a pathogenic insult (Fig. 1). Although there may be many unknown factors that contribute to these tissue-specific niches, we highlight below several that are known to or we believe are likely to impact T cell differentiation and should be considered when exploring the in vivo components shaping T cell adaptive immunity.

## INTRINSIC CELLULAR POPULATIONS THAT CONTRIBUTE TO TISSUE-SPECIFIC T CELL DIFFERENTIATION

### Dendritic cells

DCs are a heterogenous population of innate cells that can be divided into several functionally distinct groups that have been extensively described in many excellent recent reviews (36–38). Briefly, DCs can be divided into conventional or classical DCs (cDCs), plasmacytoid DCs, monocyte-derived DCs, and Langerhans cells (LCs). cDCs can be further broken into an LN resident population and a migratory population that originates in the tissue and subsequently drains to the LNs upon activation (36). Although their name implies their immovability, resident DCs can dynamically move within the LN, especially upon infection (36, 39, 40). Ag introduced via tissues can either drain directly to LNs, where it can be taken up and presented by resident cDCs, whereas migratory cDCs directly sample and acquire Ag in the tissue. Studies suggest both populations of cDCs engage in the T cell activation and differentiation process (36, 41, 42). Furthermore, both the resident and migratory cDC populations can be further divided into cDC1 and cDC2 cells. cDC1s preferentially induced Th1 responses and cross-present to CD8 T cells, whereas cDC2s preferentially induce T follicular helper and Th2 responses, although the mechanistic basis for this delineation is not entirely understood (36, 43–48). Although there are key distinctions between cDC1 and cDC2 cells, they both dynamically change upon infection or immunization. The sensing of damage- or pathogen-associated molecules leads to inflammatory cytokine upregulation, increased Ag presentation, and costimulatory signaling ligands (49). The migratory property of DCs to travel from the tissues to the LN is a critical part of DC function. Although CCR7 is a key receptor for all DC migration across tissues, tissue-specific migratory cues have also been described for skin, intestinal, and lung DCs trafficking to LN (40). It is important to note that comparison of DC populations in tissues have identified unique tissue-specific signatures associated with the tissue of origin (50). Thus, the precise tissue environment DCs originate from is likely to impact not only how DCs migrate to their draining LNs but also their precise function once there.

Many studies have explored the specific populations of DCs that migrate from peripheral tissue sites to draining LNs in the context of homeostasis, infection, and immunization with Ags or allergens (40). Broadly, the specific DC populations at each tissue site and unique functions associate with the tissue anatomy, function, and interactions with the environment. For example, within the skin, dermal DCs and LCs both can take up Ag and migrate to the draining LN; however, the kinetics by which they arrive vary, with dermal DCs arriving first and followed by LCs. In addition, the localization within the LN is altered; LCs migrate to the paracortex, whereas dermal DCs preferentially reside in the interfollicular regions (51). Yet, both LCs and dermal DCs carry Ag from commensals or pathogens where each DC population can play differential roles in promoting tolerance or induction of Th17, Th1, or Tregs, respectively (40, 46). Finally, migration of T cells back to the skin can promote additional differentiation signals from skin-resident DC populations, further underscoring the different roles DC populations can play to support tissue-specific responses (9). In the gut, Ags from commensals and food are constantly sampled by lamina propria DCs that migrate upon inflammatory signals (52, 53). Importantly, lamina propria DCs preferentially promote Treg generation via RA, likely because of the increased level of nonpathogenic Ags in the intestine that require tolerance induction (30, 31, 54). In contrast, mesenteric resident LN DCs do not have this same capacity (55, 56). More recently, the ability to promote Tregs was demonstrated to be strongest from migratory cDC1 cells in the gut because of intestinal factors such as thymic stromal lymphopoietin from epithelial cells, bile, and dietary retinoids (26, 57–59). This property can be altered in the context of inflammation, driving T cells to become Th1 cells instead, suggesting the environmental context of the gut on DC populations can influence the T cell response (23, 25, 60). Similarly, the lung has specific DC populations that respond to allergens and infection. Both cDC1s and cDC2s can take up Ag and traffic to the mediastinal LN, yet cDC2s are primarily associated with driving Th2 cells to allergens in the lung (61). More recently, inflammatory cDC2s were defined to be driven by type I IFNs and important in both allergen and viral infection for promoting T cell responses (62). Although cDC1s could promote CD8 T cell responses, inflammatory cDC2s could promote both CD8 and Th1 responses. In contrast, monocyte-derived DCs have limited migratory potential and ability to activate T cells (62, 63). More direct evidence that tissue signals can alter T cell priming in the LN was shown in a study assessing the role of TGF-β activation by migratory DCs on skin tissue-resident memory cell formation (64). DCs lacking αv integrin and unable to activate TGF-β, upon skin vaccination, were unable to form effective tissue-resident memory T cell (Trm) in the skin. This was due to epigenetic imprinting of naive CD8s in the draining LN, but not the spleen, indicating that tissue-derived DC function to activate TGF-β prior to vaccination creates a tissue-specific homeostatic niche that impacts naive T cells even before they see foreign Ag to be primed toward skin tissue-resident memory cells. Taken together, these studies of tissue-specific DC populations clearly demonstrate that DCs act as sentinels of the environment, migrate from tissues to LNs, and interact with T cells to shape their responses both in settings of homeostasis and in the context of infection.

Within the LN, there is additional organization beyond T and B cell zones that shapes the T cell response. Studies exploring DC migration have identified discrete micro niches that DCs localize to upon entry into the LN, suggesting DC subsets fulfill important functions that require their localization in specific areas of the LN, likely to interact with specific populations of immune cells (3, 65). Several studies have now shown convincingly that these DC niches play critical roles in T cell differentiation. For example, DCs positioned in the interfollicular zones located proximally to B cell zones seem to preferentially induce T follicular helper cell differentiation (66, 67). Furthermore, cDC1s and cDC2s have preferential localizations within the LN that contributes to differential location of Th1 and Th2 cells within the LN. In one recent study, type I IFNs resulted in resident DC relocalization from the periphery to the T cell zone, whereas inflammatory monocytes were recruited to form micro niches with the LN that played critical roles in early T cell priming and effector differentiation (68). Another study found that type I IFNs altered the balance of CXCL9 and CXCL10 expression on DC and stromal cell subsets that had distinct spatial locations in the LN that contributed to effector or memory T cell generation (69). Although neither study addressed specifically the role of the tissue or examined different LNs, the formation of distinct niches within the LN indicates precise gradients of chemokines and cytokines play important roles in driving effector T cell differentiation and small perturbations in those signals, possibly derived from distinct tissue sites can play a role in that process. Collectively, these studies imply that both the tissue in which the LN is draining, as well as the micro niches that form within the LN, play critical roles in shaping the T cell response. As DC populations are pivotal in both sensing changes in the environment, uptake of Ag, and migration to LNs to interact with T cells, they are likely to be highly flexible to precisely shape the T cell response needed depending on the situation.

### Stromal cells

Stromal cells play critical roles in LN organization and immune responses, as highlighted in several recent reviews (70–72). LN stromal cells comprise several populations, including fibroblastic reticular cells (FRCs), which are comprised of several subsets such as T zone reticular cells, follicular DCs that support germinal centers, marginal reticular cells, and medullary FRCs. In addition to FRCs, there are pericytes that surround blood vessels, lymphatic endothelial cells, and blood endothelial cells (70). More recent single-cell studies of stromal cell populations have indicated even more complex heterogeneity, suggesting a level of specialization that aids in compartmentalizing the immune cell niches of the LN as well as their responses to insult (73). Upon infection or immunization, LNs expand in size in large part because of changes to stromal cells that work to accommodate the rapid proliferation and expansion of T and B cells as well as infiltration of DCs, macrophages, and other immune cells (70, 74). FRC changes in response to Clec2–podoplanin interactions with DCs as well as proliferation in response to inflammatory signals such as IL-1β, IL-17, TLRs, or type I IFNs that are key in supporting LN hypertrophy and subsequent T cell responses (70). Thus, stromal cells are important players in setting the environment for immune responses.

As stromal cells are noncirculating, they are likely the best candidates for driving persistent tissue-specific signatures within an LN. Indeed, a recent study exploring stromal and other structural cells in an organ-specific manner identified gene networks that were both cell type and organ specific that shaped interactions with immune cells both at homeostasis and upon systemic infection underscoring the organ-specific nature of stromal cells and their influence on immune cells (75). More specifically, it has been well described that there is an increased frequency of Tregs in the mesenteric LNs draining the gastrointestinal tract compared with other draining LNs. Several studies exploring the mechanistic basis of this relative increase found that transplanting the mesenteric LNs to the leg of the mouse draining the footpad (popliteal fascia) retained their ability to preferentially induce Tregs even 50 wk posttransplant (34). The early presence of the microbiome after birth was required for mesenteric LNs to promote Treg induction; yet once introduced, ablation of the microbiome with antibiotics was insufficient to significantly alter the ability of the mesenteric LN to promote Tregs (76). Isolation and transcriptome analysis of stromal cells from mesenteric LN and popliteal LNs confirmed significant transcriptional differences in these two locations that was not altered upon transfer. Intriguingly, however, after transplant of mesenteric LN from germ-free mice to the leg of specific pathogen–free mice, the transcriptomes of stromal cells looked more similar to popliteal LNs than mesenteric LNs, suggesting that once imprinted by microbial components in the gut, mesenteric LN stromal cells can maintain this signature indefinitely even under different circumstances, yet unprimed stromal cells having never seen a microbiome are now capable of being modulated by a new environment. Importantly, these signatures also impacted DC populations, in particular resident DCs. Interestingly, in the study mentioned previously looking at individual LNs from the gastrointestinal tract, stromal cell (FRC and lymphatic endothelial cell) isolation and transcriptome analysis from individual gut draining LNs demonstrated gene level differences, again supporting the notion that the stromal cells play a key role in creating the homeostatic niche of individual LNs (35). Thus, these data suggest that the earliest experience of the environment is sufficient to imprint long-lasting tissue-specific signatures on stromal cells in LNs that can be maintained long term and subsequently influence T cell differentiation.

### Nervous system

The tissues are innervated by neurons that in addition to obvious roles in motor function and sensation, are also in constant communication with immune cells (77–80). The neurons in barrier tissues in particular play key roles in immune regulation, as they are constantly in contact with the environment and can directly sense pathogens and noxious stimuli (77). Although these interactions are complex and are just beginning to be understood, several studies have already highlighted the contextual nature of neuroimmune interactions on homeostasis and response to infection, suggesting tissue-specific differences may be key to subsequently shape the environment in ways that may alter downstream T cell responses.

In the skin, *Staphylococcus aureus* infection is directly sensed by TRPV1^+^ and Nav1.8^+^ nociceptor neurons by *N*-for-mylated peptides and α-hemolysin toxin. In addition to pain, this triggers release of the neuropeptide CGRP, which reduces monocyte influx, limits TNF-α production by macrophages, and limits the growth of draining LNs (81, 82). Similar mechanisms are operative in the lung in response to severe bacterial infections, in which nociceptors can suppress γδ T cells and neutrophils, limiting IL-17 responses and lethal pneumonia (83). Thus, neurons act to reduce inflammation induced by infection. In contrast, fungal infection with *Candida albicans* drives TRPV1^+^ nociceptors to induce IL-23 from CD301b^+^ dermal DCs, which subsequently act on γδ T cells to produce IL-17 required for fungal clearance (84). In the gut, enteric neurons participate in host defense by secreting IL-18, which acts on goblet cells to produce antimicrobial peptides and limit *Salmonella* infection, whereas nociceptor neurons signal via CGRP to reduce the number of M cells that are critical for invasion of *Salmonella* (85, 86). Similarly, parasitic infections of the gut trigger neuromedin U (NMU) neuropeptide production to activate ILC2s, which can work via type 2 immunity to expel the pathogen (87, 88). In contrast, CGRP expressed by nociceptor neurons or ILC2s limits ILC2 activation as part of a negative feedback loop limiting inflammation and inhibiting parasite clearance (89). Thus, the neuroimmune cross-talk in the gut both promotes host defense and protects from inflammation.

Recent studies have now extended the neuroimmune connection beyond the barrier tissues by demonstrating the presence of both peptidergic nociceptor sensory neurons and sympathetic neurons directly innervating the peripheral LNs and impacting splenic immune responses (90, 91). In one study exploring innervation of the LN, primary interactions were identified between sensory neurons and stromal cell compartments, suggesting activation of neurons likely influence LN composition and function, particularly in the context of an infection or noxious stimuli. In addition, the splenic nerve, part of the sympathetic branch of the nervous system, was required for T cell–dependent plasma cell formation to Ag (91). Choline acetyltransferase (ChAT)–expressing T cells translated norad-renergic signaling from the splenic nerve to splenic plasma cells, although the effector status of these T cells was not explored in this study. Collectively, these studies highlight that neuroimmune modulation is highly contextual across tissue sites and likely depends on the tissue, type of neuron, type of immune cell, and type of stimuli involved. As the nervous system is highly innervated in barrier tissues that are constantly in contact with the environment as well peripheral LNs, neurons likely constantly signal to the immune system to either suppress immune responses and maintain homeostasis or promote inflammation in the context of pathogen insult. In addition, they can limit inflammation in the context of infection to aid in restoration of homeostasis. Therefore, it is highly likely that neuroimmune interactions play key roles in promoting signals that contribute to T cell differentiation and effector function; however, the specific interactions between neurons and T cells are limited, and this is an area of research that is likely to shape our future understanding of T cell differentiation and function in the future.

## NONCELLULAR EXTERNAL FACTORS THAT CONTRIBUTE TO TISSUE-SPECIFIC BIOLOGY

### Metabolism

In addition to the tissue-specific cellular populations described above that can impact T cell differentiation, many external factors can be present and influence in the tissue space. Alterations in metabolism are now well understood to be tissue specific, and the role of metabolism on T cell differentiation and function has more recently come to be appreciated as described in several reviews (92–96). Indeed, T cells can shift their metabolic capacity in settings of nutrient restriction or in an altered metabolic tissue environment to adapt accordingly. As T cells can migrate throughout the body, they must be capable of sensing changes in the metabolic environment and subsequently adapt (96). These adaptations can include alterations of effector cell differentiation as well as survival and proliferation of their effector state. Early after activation, T cells shift their metabolic state from mitochondrial oxidative phosphorylation to aerobic glycolysis (97). In contrast, Tregs prefer fatty acid oxidation even in a setting with excess glucose (93, 98). Recent studies have profiled the metabolic state and capacity of T cells in a variety of settings. Although many in vitro studies have outlined key pathways controlling metabolic functions of T cells, evidence suggests in vivo things are more complicated (99). The metabolic state of tissues is also unique and dynamic, with varying combinations of nutrients and signals that T cells must adapt to to proliferate, differentiate, and survive. Changes in the metabolic niche can have significant impacts on T cells and can alter activation, survival, or effector differentiation (100).

Mitochondrial metabolism is critical for T cell activation and a key component of function (101). Although broadly thought to switch to aerobic glycolysis upon activation, effector T cell reliance on oxidative phosphorylation in vivo maybe more heterogeneous than results described in vitro, suggesting adaptation to tissue environments might be key in controlling this heterogeneity (99). In addition, differentiation of effector CD4s use disparate components of the electron transport chain, as Th17 cells more heavily use complexes I and II, whereas Th1 cells required active complexes II and III (102). Limiting oxidative phosphorylation inhibits Th17 cells and alternatively promotes Tregs (103). Finally, memory cells rely on mitochondrial metabolism for their formation and maintenance as well as shift to use lipolysis and glycerol to maintain their survival (104, 105). In tumors, mitochondrial dysfunction driven by environmental signals such as continuous Ag stimulation and hypoxia promote loss of effector function (106–108). Thus, T cell differentiation and function rely on mitochondrial metabolism, and the tissue environments can play important roles controlling the availability of metabolic inputs to fuel metabolism of the cells.

There are several metabolic conditions present in a variety of settings in which T cells have evolved mechanisms of adaptation. One common metabolic state is that of low glucose and high lactate, which is often found in solid tumors, inflamed tissues such as arthritic joints, atherosclerotic plaques, and in the adipose tissue of obese people. These inflammatory settings are ones without pathogenic initiation and tend to be long-term states of low-grade chronic inflammation. T cells in these environments of low glucose and high lactate undergo metabolic adaptations to use lactate rather than glucose for metabolic survival. Tregs upregulate genes involved in lactate metabolism, enabling them to convert lactic acid into pyruvate, inhibiting aerobic glycolysis and supporting the metabolic preference for oxidative phosphorylation in Tregs, allowing them to maintain their suppressive function (109–111). In contrast to Tregs, effector T cells are inhibited in the low-glucose and high-lactate setting of tumors, leading to reduced T cell activity (112–114). Yet, in the high-lactate conditions in settings of inflamed joints and after increased exercise regimens, effector T cells could increase their capacity to endure high lactic acid concentrations via upregulation of lactate transporters or changes to their central carbon metabolism and remain functional or increase their functional capacity to clear tumors (115, 116). Thus, for effector T cells, the context in which the cells experience high lactate may play a key role in whether function is inhibited or if cells can rewire and adapt to their changing environment.

Hypoxia is another common state that T cells have mechanisms to adapt. Hypoxia, or settings of low oxygen availability, activate a specific transcription factor in T cells called hypoxia-induced factor 1 α (HIF-1a) (117). In settings of normoxia, oxygen-sensing proteins (PHD containing) inhibit HIF-1a by marking it for degradation via ubiquitination. When oxygen levels become low, HIF-1a becomes stable, translocates to the nucleus, and activates target genes and transcriptional pathways that allow T cells to survive and function in the hypoxic environment. Loss of Vhl, the negative regulator of Hif1a in CD8 T cells, leads to unrestrained effector cell differentiation, leading to excessive immunopathology and death postinfection of chronic virus (118). Increased glycolysis and effector molecules suggested that in settings of hypoxia in which cells are nutrient limited, HIF-1a serves to boost metabolic pathways to meet energy demands and impact important effector molecules (118). Hypoxia is often found in tumors in which glucose levels are low because of the high glycolytic needs of the tumor itself. In tumors, hypoxia promotes metabolic dysfunction of T cells by driving reactive oxygen species leading to increased phosphotyrosine signaling and NFAT localization to the nucleus and subsequent T cell exhaustion (106). Thus, although T cells can adapt to hypoxic environments, altered nutrient environments can drive T cells into a state in which their function is somewhat limited, suggesting multiple adaptations of T cell exist to maintain survival in harsh metabolic environments.

Although changes in metabolism to meet the energetic demands of the cell appear obvious, a less-appreciated, but likely important, role for cellular metabolism are the metabolic byproducts needed for epigenetic changes upon T cell activation. Changes to metabolic pathways have consequences for the availability of key metabolites, which are required for epigenetic modifications and transcription (119). The glycolytic cycle of activated T cells results in increased levels of acetyl-CoA and α-ketoglutarate. Glucose fuels the majority of acetyl-CoA production in proliferating cells, which is largely responsible for acetylation of cellular proteins, including histones (120). α-Ketoglutarate, another metabolite produced by glycolysis, is required for function of both DNA and histone demethylases (121). *S*-adenosylmethionine, a product of one-carbon metabolism, is a methyl donor for methyltransferases. Therefore, there are many ways in which metabolic pathways of proliferating cells intersect with epigenetic changes in cells. For T cells, this is likely to be critical. Upon activation, T cells undergo rapid blasting and differentiation, indicating changes to the epigenome are key in taking on the fate and function dictated by the environment. Yet, although the role metabolites play in the epigenome are clear, as is the requirement for metabolic shifts upon T cell activation, very few studies have linked the metabolic changes with the precise epigenetic changes that are required for T cell effector differentiation. This has been particularly difficult to study in vivo in which metabolic changes in the tissues may be the most important for linking metabolic changes to epigenetic effects that impact the differentiation and function of T cells. Therefore, we hypothesize that tissue-specific metabolic environments may play fundamentally key roles in changing the differentiation and function of T cells in a potentially heritable manner, as changes that occur to the epigenome has the power to be passed to future generations of cells.

### Microbiome

The microbiome is an important part of mammalian health and survival. As mammals evolved with microbes, a symbiotic relationship was established in which microbial communities are important for tissue development, metabolism, and host defense (122). Importantly, the gastrointestinal tract, skin, lung, oral cavity, and vaginal cavity each have completely unique microbial communities. In fact, niches within the skin, such as hair follicles, as well as each distinct portions of the gut have distinct microbial communities. These communities are also dynamic, and changes in diet, habitat, systemic infection, or antibiotic use have been known to shift those communities (10). The diversity of the microbiome across tissue spaces implies that there is local tissue compartmentalization and specialization controlled by both the host and the microbiome that is key both during homeostasis and under pressure from local inflammatory responses.

The ability of the microbiome to influence T cell differentiation is evident in many studies that have assessed the T cell response to the microbiome (123). The gastrointestinal tract bears the largest microbiome in terms of bacterial number, as well as in the diversity of species. In the lamina propria of the intestine, up to 30–40% of T cells may be Th17 cells; their production of IL-17A, IL-17F, and IL-22 contributes to the production of antimicrobial peptides by intestinal epithelial cells, the maintenance of tight junctions that maintains the barrier function of the epithelium and the secretion of IgA (124, 125). Germ-free mice have limited Th17 cells in the intestine as well as the skin, confirming the critical role of the microbiome in promoting Th17 cells (8, 126, 127). One key species involved in the development of Th17 cells in the gut is SFB (128). The presence of SFB directly correlates with levels of Th17 cells in the gut; however, the initiation of this pathway occurs in the mesenteric LN (129). Monocyte-derived CX3CR1^+^ cells respond to environmental factors driven by SFB to active ILC3s, which can promote Th17 cell responses (130). In the mesenteric LN, T cells upregulate Rorγt, and upon trafficking back to the intestinal tissue, they can secrete IL-17 and IL-22 in response to local tissue production of serum amyloid A proteins 1 and 2 (SAAl/2) driven by ILC3 cells (129, 131). Thus, the presence of a single microbe, SFB, is sufficient to promote an environment unique to the gut that impacts T cell activation in the local draining LN. A similar mechanism is operative in the skin, where the commensal bacteria S. *epidermidis* is critical for Th17 and IL-17–producing CD8 T cells in the skin (9). Alterations in the gut microbiome do not play a role in shaping the T cell response to S. *epidermidis*, suggesting the local microbiome is shaping the local T cell response.

Tregs are also common in the intestine, where they play a key role in maintaining tolerance to the microbiome. Similar to Th17 cells, germ-free mice are largely devoid of intestinal Tregs, implicating the microbiome in their induction. TGF-β and RA also play a key role in driving Tregs in the gut. Intriguingly, many Tregs in the intestine express Rorγt, suggesting the specific environment signals of the intestine are optimal for induction of specific programs in T cells. Specifically, IL-6 and RA in the intestine are key for the induction of Rorγt, induced via the presence of a microbiome (132). More recently a role for diet and microbiome influenced colonic bile acids have been shown to induce Rorγt in Tregs, and IL-6 can be produced by enteric nerves in the colonic lamina propria that are heightened in the absence of commensals, leading to an increase in Rorγt Tregs (133, 134). Alternatively, a Gata3^+^ population of Tregs also exists in the intestine that depends on the presence of IL-33 (135, 136). Unlike SFB, the precise microbial species that induce Tregs is less clear. Clostridia strains have been shown to induce Tregs in several settings, possibly by the induction of short-chain fatty acids (SCFA), for which Clostridia contain several genes predicted to be capable biosynthesis of SCFA (137). SCFA are natural HDAC inhibitors and thus suppress proinflammatory cytokine production of DCs. Several other bacterial strains have been linked to Treg induction in the intestine, and although the precise mechanism of each is still unclear, the overarching picture is that microbial components create an environment that is key to generating a tolerogenic state that includes the induction of Tregs and Th17 cells. Importantly, in the setting of systemic inflammation such as viral or parasitic infection, these tolerogenic signals can be overridden and microbiome-specific T cells converted into proinflammatory subsets such as Th1 cells (23). Thus, the tissue environment is mutable and can be modified to accommodate shifts in the environment to produce the appropriate output needed.

In comparison with the intestine or skin, the lung microbiota is vastly smaller in size; however, there is bacteria present in the lung that, like the gut and skin, is colonized early in the neonatal period (138). The benefit of the lung microbiome has been primarily studied in the context of allergic asthma, in which the presence of a microbiome confers some protection toward the development of allergic asthma, likely by driving Tregs, a similar mechanism as the intestine and skin (139–141). The precise nature of this effect is not entirely clear, as depending on the model of allergic asthma used, the level of protection afforded is different (139, 141). Yet, a collection of studies together clearly show that the presence of a lung microbiome drives Tregs in early life, which is likely to create a tolerogenic environment that limits inflammation to allergens (142). Furthermore, several lung inflammatory diseases have altered microbiomes, suggesting immune dysregulation leads to changes in microbial diversity and further suggests the constant role of the immune system to sense and respond to the micro-biome in the lung (138). It is important to note, however, that these spaces are not entirely isolated from one another. There has been clear demonstration of a gut–lung axis in both humans and mice, as changes in the intestinal microenvironment has impacts on lung disease (142–145). Furthermore, the immune-associated lymphoid tissues of the lung and gut (inducible BALT and GALT) have similarities both in their function and morphology, suggesting cells can migrate between the tissues. Although the precise mechanisms establishing and controlling the gut–lung axis remain to be completely defined, the factors shaping one tissue can, in some ways, lead to alterations in distant tissues in a coordinated fashion, further underscoring the dynamic nature of the immune system with the changing environment.

Taken together, the interplay of the microbiome with the immune system at barrier surfaces is undeniable, thus microbial Ags are constantly sampled at each tissue site, helping to shape the tissue-specific responses that impact downstream adaptive immune pathways. Given the requirement of the microbiome for proper immune tolerance at barrier sites and the dysregulation that occurs in the context of inflammatory disease, it is clear that the diversity of the microbiome at the tissues plays a key role in shaping the homeostatic niche found at each site. As the precise bacterial Ags vary by tissue space and potentially in response to changes in diet, antibiotic use, etc., the nature of the response will be varied as well, thus creating tissue-specific homeostatic niches that are key to setting the tone of each tissue as well as creating the environments in which T cells will become activated and differentiate.

### Foreign Ags

In addition to tolerance to self-Ags and to microbial Ags described above, the nonpathogenic environmental Ags we encounter daily also require homeostasis. What we eat, what we breathe, and what we touch represent many additional foreign Ags introduced via tissue sites that the immune system must recognize and generate homeostasis. And yet, for a sizable portion of the population, tolerance is not intact—inappropriate responses to nonmicrobial environmental Ags are the primary cause of allergic responses at multiple tissue sites and are on the rise in both children and adults. Food allergy, skin allergies such as atopic dermatitis, and allergic asthma are several inflammatory responses at tissue sites that play important roles in morbidity and rising health care costs. In some cases, responses are focused at the primary tissue site of allergen interaction, for example allergic asthma to inhaled allergens, such as house dust mite. Yet, in most cases, allergies can drive multitissue responses such as hives or anaphylaxis, particularly upon secondary encounter. T cell–mediated IgE responses to allergens are thought to largely drive systemic or distal tissue responses, but initial sensitization of allergens likely occurs in a tissue-specific manner. Tolerance mechanisms such as DCs and Tregs at the tissue site constantly survey and limit immune reactions to nonpathogenic Ags in a similar manner as microbial commensal Ags. Tissue-located DCs constantly sample the local Ags and drive Tregs to environmental Ags such as food to limit any aberrant effector responses that may occur. As each tissue site experiences different Ags, local environmental diversity in Ag exposure likely creates an impression of the tissue in the draining LN, creating slightly different homeostatic environments at each LN that may impact immune responses similar to that of the microbiome, as discussed above. In the gut, mechanisms similar to microbial Ag detection are operative: DCs expressing CX3CR1 and CD11b sample luminal contents to capture Ag (146), whereas direct Ag transfer can occur through specialized epithelial cells such as M cells (147). Several factors play a key role in driving tolerance within the mesenteric LN, such as TGF-β, RA, IDO, and IL-10. Both Tregs and non-Foxp3^+^ Tregs that produce IL-10 maintain tolerance to environmental Ags, as loss of Tregs will reverse established tolerance (148). The skin has similar mechanisms, in which a specialized DC population called LCs as well as dermal DCs sample Ag in an immature or tolerogenic state and traffic to the draining LN to induce T cell responses (149). The skin is also rich in Trms, which do not circulate, and under homeostatic conditions, many Trms are Tregs or a Tr1 cell that produces IL-10 and TGF-β (150). Largely similar mechanisms exist in the lung, where Ag is sampled by macrophages and lung DCs and transported to LNs, and drive the production of Tregs, and regulatory tissue-resident memory cells are found in the lung tissue as well. IL-10, TGF-β, and RA are all key players in lung tolerance mechanisms. The circulation of Ag, either carried by DCs or more freely transported, plays a key role in establishing tolerance via induction of Tregs. Therefore, together with the microbiome, the local environmental Ags encountered at each tissue site may play a key role in creating tissue-specific responses both at the tissue site and within the LNs draining those tissues, which is likely to play an important role in setting the environmental tone present when T cells see Ag to become activated and begin their differentiation process.

## CONCLUSIONS

The notion of tissue-specific immunity is widely accepted. Each tissue has specific functional needs and is also experiencing the surrounding environment based to some extent on those tissue-specific functions. For example, the mucosal tissues such as the lung and gut have specific differences from the skin based on its mucosal nature. As such, the immune system reacts in a tissue-specific manner as well, controlling tolerance to the environment while also mounting responses to foreign pathogens if needed. These tissue-specific environmental changes impact the immune cells in the tissue directly as well as those that exist in the draining LN, setting up local neighborhoods in which the LN has tissue-specific features that make it optimally primed to generate a response that is needed for the tissue in which Ag is encountered. There is ample evidence that immune responses to similar Ags have widely different outcomes depending on the site of Ag introduction, and exploration of cells in the tissues and draining LNs confirm that APCs such as DCs and stromal cells have transcriptomes consistent with local imprinting from the tissue. Furthermore, specific neuron populations at each tissue site are likely to have an impact on T cell activation and effector function, even if that interaction is indirect. We argue therefore that these many variables, both intrinsic cellular populations in the tissue and LN spaces as well as external factors such as the metabolism of the tissue or microbiome, can have an important impact on the process of T cell differentiation in vivo. So much of our knowledge on T cell activation and effector function in vivo is limited because of technical limitations; however, new in vivo and ex vivo assays should allow us to revisit the factors controlling T cell differentiation and function to more precisely understand the key tissue-specific factors that are important for controlling responses to infection through the lens of tissue-specific immunity.

## Abbreviations:

cDC: classical DC
DC: dendritic cell
FRC: fibroblastic reticular cell
HIF-1a: hypoxia-induced factor 1 α
LC: Langerhans cell
LN: lymph node
RA: retinoic acid
SCFA: short-chain fatty acid
SFB: segmented filamentous bacteria
Treg: regulatory T cell
Trm: tissue-resident memory T cell
